# Imaging enzyme kinetics at atomic resolution

**DOI:** 10.1107/S2052252516010204

**Published:** 2016-06-27

**Authors:** John Spence, Eaton Lattman

**Affiliations:** a Physics, Arizona State University, Rural Rd, Tempe, 85287-1504, USA; b Hauptman-Woodward Medical Research Institute, 700 Ellicott Street, Buffalo, NY 14203-1101, USA

**Keywords:** serial crystallography, catalysis, enzyme mechanism, denitrification, radiation damage, radiolysis, synchrotron radiation

## Abstract

Serial crystallography at a synchrotron has been used to obtain time-resolved atomic resolution density maps of enzyme catalysis in copper nitrite reductase. Similar XFEL studies, intended to out-run radiation damage, will also soon appear.

The 1958 Nobel prize to Beadle and Tatum for proposing that each gene is responsible for a distinct enzyme is now seen as both foundational to molecular biology and genetics, albeit oversimplified. Some genes, for example, code for functional RNAs, while others code for non-enzymatic proteins such as collagen. Yet enzymes remain fundamental to life on earth, catalyzing at least 5000 biochemical reactions (so far identified). Enzymes can increase reaction rates by huge factors, from millions of years to milliseconds per event, so that, from meat tenderizer to washing powder, to muscle contraction, cargo transport in the cell, ion pumps, infection and digestion, no molecular machine is more fundamental to biological function than the enzyme.

How do they work? Fischer’s 1894 ‘lock and key’ model, establishing the enzyme and substrate model, was improved by Koshland to include induced fit and molecular recognition, and it has long been understood that enzymes lower the Gibbs free (activation) energy by stabilizing an intermediate state, providing an alternative reaction pathway, or by destabilizing the substrate ground state, all of which can now be understood in terms of an energy landscape. Michaelis and Menten first proposed the kinetics for a two-step model for conversion of substrate to product. Allostery, feedback, inhibitors (including drugs and poisons) and activators are all now known to be important in enzyme regulation.

The prospect of imaging such molecular machines during their catalytic cycles by a kind of atomic resolution X-ray molecular movie is brought a step closer in this issue in the paper by Horrell *et al.* (2016 ▸). Their approach is based on a variant of ‘serial crystallography’, a new approach to crystallography developed for X-ray lasers (Spence & Doak, 2004 ▸; Shapiro *et al.*, 2008 ▸; Chapman *et al.*, 2011 ▸) and now increasingly popular at synchrotrons (Nogly *et al.*, 2015 ▸). There, a continuous stream of hydrated bioparticles, such as protein nanocrystals, flows across the X-ray beam in single file but with random orientations. Particle diffraction conditions and orientation are analyzed later by smart algorithms. Horrell *et al.* soaked ‘large’ crystals (perhaps 100 000 times larger in volume than the micrometre-sized crystals used at X-ray lasers) of recombinant copper nitrite reductase in sodium nitrite for an hour at room temperature before transferring them to a cryoprotectant and plunging into liquid nitro­gen, to trap the structure of the room temperature complex. At this stage the reaction does not proceed because no reducing agent is present. The required electrons are provided by free radicals generated by the very X-rays used to image the structure.

The authors then used the Diamond synchrotron fitted with a new fast shutterless detector to obtain 45 low-dose Bragg diffraction datasets in 19 s each from the same regions of the crystal extending to almost 1 Å resolution. This interval spans the catalytic cycle of nitrite reduction, a vital process in agriculture and in the formation of the greenhouse gas N_2_O.

Previous observations of radiation-induced reactions in protein crystallography include those devoted to horseradish peroxidase (Berglund *et al.*, 2002 ▸). Studies have also shown that in many cases enzymes remain active in crystalline form. In all cases, one must show that the effect of electron ionization by the weak X-ray beam induces the catalytic reaction, rather than damaging the crystal. In this work, Horrell *et al.* find little reduction in resolution during the collection of their low-dose ‘molecular movie’, in which the time-resolved density maps do show conversion of substrate at a catalytic copper center from nitrite to nitric oxide, suggesting (along with analysis of data collection statistics) that they are seeing chemistry relevant to catalysis, not predominantly radiation damage.

Thus the new fast synchrotron detectors (soon to be combined with diffraction-limited sources) may allow us to make movies of enzyme kinetics. The interpretation of these is not straighforward, since from a crystal of reacting molecules one reconstructs a spatially periodic average density map of the sum of all stable intermediate states as a function of time, representing the total kinetics, rather than the dynamics of molecular transformations at the atomic scale. The latter occur too rarely and rapidly to be imaged or even simulated on the correct timescale. It remains to be seen if the often-cited advantage of EXFELs to ‘outrun’ radiation damage, using brief pulses instead of sample cooling, will allow imaging at the physiological temperatures which provide the correct energy for the reaction, and so provide a better approach, also based on serial crystallography. Studies of enzyme dynamics have been proposed at EXFELs (Schmidt, 2013 ▸; Wang *et al.*, 2014 ▸) and are now under way, while pump-probe studies of light-sensitive proteins have recently provided ‘movies’ at near-atomic resolution (Pande *et al.*, 2016 ▸). Thus optogenetics may offer a way forward for triggering these reactions, even in enzymes (Moffat, 2014 ▸). If these methods can be used to assist in modelling the atomistic mechanisms for enzymatic catalysis, it may indeed be possible, using recombinant DNA, to develop new enzymes with wanted properties, with huge implications for pharmacology, drug treatment and food production, among other fields of biochemistry.

## References

[bb1] Berglund, G., Carlsson, G. H., Smith, A. T., Szöke, H., Henriksen, A. & Hajdu, J. (2002). *Nature*, **417**, 463–468. 10.1038/417463a 12024218

[bb2] Chapman, H., Fromme, P., Barty, A., White, T. A., Kirian, R. A., Aquila, A., Hunter, M. S., Schulz, J., DePonte, D. P., Weierstall, U., Doak, R. B., Maia, F. R. N. C., Martin, A. V., Schlichting, I., Lomb, L., Coppola, N., Shoeman, R. L., Epp, S. W., Hartmann, R., Rolles, D., Rudenko, A., Foucar, L., Kimmel, N., Weidenspointner, G., Holl, P., Liang, M., Barthelmess, M., Caleman, C., Boutet, S., Bogan, M. J., Krzywinski, J., Bostedt, C., Bajt, S., Gumprecht, L., Rudek, B., Erk, B., Schmidt, C., Hömke, A., Reich, C., Pietschner, D., Strüder, L., Hauser, G., Gorke, H., Ullrich, J., Herrmann, S., Schaller, G., Schopper, F., Soltau, H., Kühnel, K., Messerschmidt, M., Bozek, J. D., Hau-Riege, S. P., Frank, M., Hampton, C. Y., Sierra, R. G., Starodub, D., Williams, G. J., Hajdu, J., Timneanu, N., Seibert, M. M., Andreasson, J., Rocker, A., Jönsson, O., Svenda, M., Stern, S., Nass, K., Andritschke, R., Schröter, C., Krasniqi, F., Bott, M., Schmidt, K. E., Wang, X., Grotjohann, I., Holton, J. M., Barends, T. R. M., Neutze, R., Marchesini, S., Fromme, R., Schorb, S., Rupp, D., Adolph, M., Gorkhover, T., Andersson, I., Hirsemann, H., Potdevin, G., Graafsma, H., Nilsson, B. & Spence, J. C. H. (2011). *Nature*, **470**, 73–77.

[bb10] Horrell, S., Antonyuk, S. V., Eady, R. R., Hasnain, S. S., Hough, M. A. & Strange, R. W. (2016). *IUCrJ* **3**, 271–281. 10.1107/S205225251600823X PMC4937782 27437114

[bb3] Moffat, K. (2014). *Philos. Trans. R. Soc. B: Biol. Sci.* **369**, 20130568. 10.1098/rstb.2013.0568 PMC4052877 24914168

[bb4] Nogly, P., James, D., Wang, D., White, T. A., Zatsepin, N., Shilova, A., Nelson, G., Liu, H., Johansson, L., Heymann, M., Jaeger, K., Metz, M., Wickstrand, C., Wu, W., Båth, P., Berntsen, P., Oberthuer, D., Panneels, V., Cherezov, V., Chapman, H., Schertler, G., Neutze, R., Spence, J., Moraes, I., Burghammer, M., Standfuss, J. & Weierstall, U. (2015). *IUCrJ*, **2**, 168–176. 10.1107/S2052252514026487 PMC4392771 25866654

[bb5] Pande, K., Hutchison, C. D. M., Groenhof, G., Aquila, A., Robinson, J. S., Tenboer, J., Basu, S., Boutet, S., DePonte, D. P., Liang, M., White, T. A., Zatsepin, N. A., Yefanov, O., Morozov, D., Oberthuer, D., Gati, C., Subramanian, G., James, D., Zhao, Y., Koralek, J., Brayshaw, J., Kupitz, C., Conrad, C., Roy-Chowdhury, S., Coe, J. D., Metz, M., Xavier, P. L., Grant, T. D., Koglin, J. E., Ketawala, G., Fromme, R., rajer, V., Henning, R., Spence, J. C. H., Ourmazd, A., Schwander, P., Weierstall, U., Frank, M., Fromme, P., Barty, A., Chapman, H. N., Moffat, K., van Thor, J. J. & Schmidt, M. (2016). *Science*, **352**, 725–729.

[bb6] Schmidt, M. (2013). *Adv. Cond. Matt. Phys.* **2013**, 167276.

[bb7] Shapiro, D. A., Chapman, H. N., DePonte, D., Doak, R. B., Fromme, P., Hembree, G., Hunter, M., Marchesini, S., Schmidt, K., Spence, J., Starodub, D. & Weierstall, U. (2008). *J. Synchrotron Rad.* **15**, 593–599. 10.1107/S0909049508024151 18955765

[bb8] Spence, J. & Doak, R. B. (2004). *Phys. Rev. Lett.* **92**, 198102. 10.1103/PhysRevLett.92.198102 15169448

[bb9] Wang, D., Weierstall, U., Pollack, L. & Spence, J. (2014). *J. Synchrotron Rad.* **21**, 1364–1366. 10.1107/S160057751401858X PMC4211133 25343806

